# Exploring the potential of naturally occurring antimicrobials for managing orthopedic-device-related infections

**DOI:** 10.5194/jbji-9-249-2024

**Published:** 2024-10-31

**Authors:** Baixing Chen, T. Fintan Moriarty, Hans Steenackers, Georges F. Vles, Jolien Onsea, Thijs Vackier, Isabel Spriet, Rob Lavigne, R. Geoff Richards, Willem-Jan Metsemakers

**Affiliations:** 1 Department of Trauma Surgery, University Hospitals Leuven, Leuven, Belgium; 2 Department of Development and Regeneration, KU Leuven, Leuven, Belgium; 3 AO Research Institute Davos, Davos, Switzerland; 4 Department of Microbial and Molecular Systems, Centre of Microbial and Plant Genetics (CMPG), KU Leuven, Leuven, Belgium; 5 Department of Orthopaedic Surgery, University Hospitals Leuven, Leuven, Belgium; 6 Institute for Orthopaedic Research and Training (IORT), KU Leuven, Leuven, Belgium; 7 Pharmacy Department, University Hospitals Leuven, Leuven, Belgium; 8 Pharmacotherapy, Department of Pharmaceutical and Pharmacological Sciences, KU Leuven, Belgium; 9 Laboratory of Gene Technology, Department of Biosystems, KU Leuven, Leuven, Belgium

## Abstract

Orthopedic-device-related infections (ODRIs) are challenging clinical complications that are often exacerbated by antibiotic resistance and biofilm formation. This review explores the efficacy of naturally occurring antimicrobials – including agents sourced from bacteria, fungi, viruses, animals, plants and minerals – against pathogens common in ODRIs. The limitations of traditional antibiotic agents are presented, and innovative naturally occurring antimicrobials, such as bacteriophage therapy and antimicrobial peptides, are evaluated with respect to their interaction with conventional antibiotics and antibiofilm efficacy. The integration of these natural agents into clinical practice could revolutionize ODRI treatment strategies, offering effective alternatives to conventional antibiotics and mitigating resistance development. However, the translation of these compounds from research into the clinic may require the substantial investment of intellectual and financial resources.

## 1 Introduction

The use of orthopedic implants has greatly benefited human health, enabling a rapid recovery for a wide range of patients (Moriarty et al., 2012). Nevertheless, infectious complications, collectively hereafter referred to as orthopedic-device-related infections (ODRIs), can lead to an impaired quality of life, challenges for the medical team and high costs for society (Moriarty et al., 2016). ODRIs encompass both periprosthetic joint infections (PJIs) and fracture-related infections (FRIs). The total number of PJIs has been on the rise in recent years, ranging between 0.5 % and 2.0 % for knee replacements and between 0.5 % and 1.0 % for hip replacements, primarily due to the increasing number of high-risk patients, including those using immunosuppressants, undergoing chemotherapy or affected by obesity (Patel et al., 2023). The incidence of FRIs is estimated at 1 %–2 % for closed fractures and up to 30 % for severe open injuries (Moriarty et al., 2022). Furthermore, with the growing number of surgically managed fractures, it is expected that the absolute number of FRIs will also rise in the future.

The challenge in treating ODRIs is often attributed to antimicrobial resistance (AMR), the formation of biofilm on the surface of the implant and within the bone, the limited penetration of antibiotics to the infection site, and the presence of abscesses that can further complicate treatment (Hofstee et al., 2021). Treatment algorithms for ODRIs usually involve surgical debridement, with removal or exchange of the implant in some cases, followed by long-term antibiotic treatment (Depypere et al., 2020). However, when treatment does not involve implant removal, treatment failure has been reported to occur in 45 % of PJI cases (Lora-Tamayo et al., 2013) and 21.4 % of FRI cases (McNally et al., 2022). Furthermore, treatment failure rates increase in patients with highly drug-resistant pathogens (Giannitsioti et al., 2022), including methicillin-resistant *Staphylococcus aureus* (MRSA) and multidrug-resistant (MDR) *Pseudomonas aeruginosa* (Jamei et al., 2017). There has been a concerning rise in antibiotic resistance among strains that commonly infect patients with orthopedic implants, such as *S. aureus* and *S. epidermidis*, and a notable increase from 9 % to 16 % in MDR Gram-negative bacilli causing PJI in the past decade (Benito et al., 2016). Moreover, biofilms create a protective environment in which bacteria can grow and persist without being subjected to the patient's immune response or antibacterial treatment. Implant surfaces are particularly favorable for biofilm formation for several reasons: they provide a surface for microbial attachment, reduce access to immune cells and cause a reduced immune response to microbes due to immune system exhaustion from the inflammatory reaction to the implant itself (Hoiby et al., 2010; Yokogawa et al., 2018).

**Figure 1:**
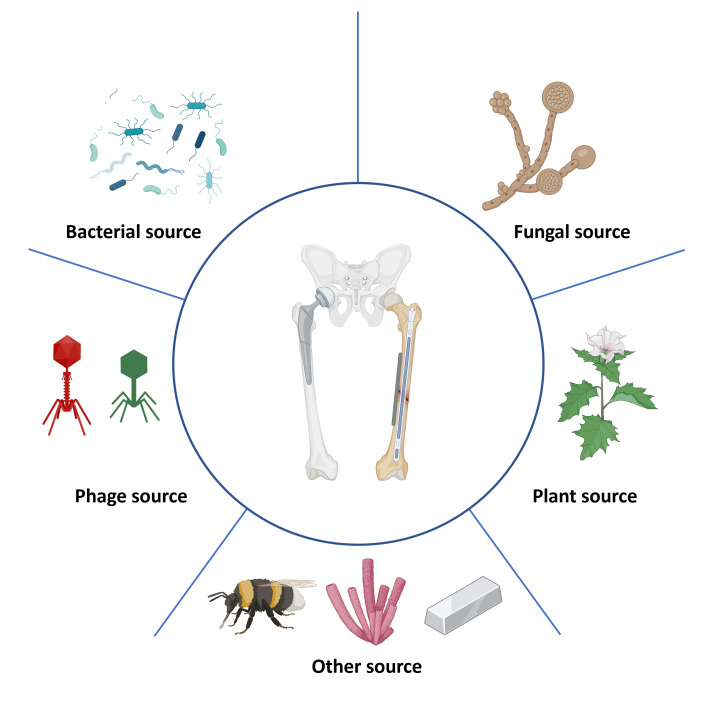
Naturally occurring antimicrobials targeting orthopedic-device-related infections. This figure depicts a range of naturally occurring antimicrobial agents that have potential applications in combating infections related to orthopedic devices. Bacterial sources are illustrated by various bacteria that produce antimicrobial substances. Fungal sources are indicated by structures resembling fungi that produce antibiotics. Viral sources are represented by bacteriophages that specifically infect bacteria, offering a targeted approach to killing bacterial pathogens. Plant-derived sources are represented by a plant, showcasing natural compounds like alkaloids, flavonoids, or essential oils with antimicrobial properties. Other sources involved are insect-derived antimicrobials, marine-derived antimicrobials and metal-based antimicrobials.

As we are confronted with the growing challenges of AMR and biofilm formation in ODRIs, it becomes crucial to explore alternative treatment strategies. Naturally occurring antimicrobials include those present in or derived from plant or animal tissues and those produced by microorganisms. These natural compounds are often part of the organisms' defense mechanisms, evolved over millennia, providing a robust and sustainable option for combating AMR and biofilm formation. Naturally occurring antimicrobials, derived from bacteria, fungi, viruses, animals and plants, present a promising avenue due to their unique mechanisms of action and lower propensity for inducing resistance compared with synthetic antimicrobials (Fig. 1). This narrative review provides a descriptive overview of the multifaceted realm of naturally occurring antimicrobials and their pivotal role in addressing the escalating challenges posed by ODRIs.

## 2 Antimicrobials derived from bacteria and fungi

### 2.1 Approved antibacterial drugs derived from bacteria and fungi

The discovery of penicillin, produced by *Penicillium chrysogenum*, as an effective antistaphylococcal antibiotic has opened the door for the development of multiple antibiotics from fungal sources, like *Cephalosporium acremonium* (cephalosporins), as well as from bacterial origins, such as *Streptomyces* (e.g., streptomycin, vancomycin and tetracycline) and *Bacillus polymyxa* (colistin). These antibiotics, discovered between 1920 and 1950, have played a pivotal role in the prevention and treatment of ODRIs. Despite their historical significance, the rapid rise in antibiotic resistance has become a serious concern that compromises the efficacy of these conventional antibiotics (Campoccia et al., 2006). Notably, even potent antibiotics like vancomycin, derived from *Streptomyces*, and cefepime, developed from the fungus *Acremonium*, which have been effective against infections caused by MDR pathogens, are encountering challenges. For instance, while still relatively rare, clinical isolates of *S. aureus* demonstrating intermediate and, in some cases, complete resistance to vancomycin have emerged over recent years. These cases, although infrequent, with a prevalence of less than 1 % among clinical isolates, represent a significant public health concern due to the critical role that vancomycin plays in treating severe bacterial infections (Shariati et al., 2020).

### 2.2 Antibacterial drugs derived from bacteria and fungi in development

The majority of new drugs approaching the final stages of development are typically derived from bacteria and fungi and tend to fall within existing antibiotic classes (Table 1). These new antibiotics have shown varying degrees of effectiveness against antibiotic-resistant bacteria in clinical and laboratory settings. The extent of their testing against resistant strains varies, with some already having undergone extensive clinical trials and others still in earlier phases of testing. To address the problem of AMR, it is important to develop new metabolites that target novel bacterial-resistance mechanisms. Most antibiotics in clinical trials are derived from a limited number of microbes, with only a few coming from new classes of microbes (Table 1; Butler and Paterson, 2020). One innovative class involves pleuromutilin, derived from the basidiomycete fungus *Pleurotus mutilus*, which demonstrates a broad spectrum of antimicrobial activity (Mapook et al., 2022). Lefamulin, a distinct member of the pleuromutilin class, acts by binding to highly conserved ribosomal targets to inhibit the binding of transfer RNA, disrupting bacterial protein synthesis. Unlike tetracyclines, which inhibit tRNA binding to the ribosome by binding to the 30S ribosomal subunit, lefamulin targets the 50S ribosomal subunit. This unique binding site allows lefamulin to interfere with the peptidyl transferase center, a crucial part of the ribosome responsible for peptide bond formation during protein synthesis. Notably, its mechanism of action differs from existing protein synthesis inhibitors, such as macrolides, reducing the likelihood of cross-resistance and lowering the potential for resistance development (Veve and Wagner, 2018). In a clinical study on acute bacterial skin and skin structure infections caused by Gram-positive pathogens, lefamulin demonstrated comparable clinical success rates to vancomycin, marking an initial proof of concept for pleuromutilin antibiotics in this context. Lysostaphin, an enzyme from *S. simulans* was studied as an antistaphylococcal agent in the 1970s but faced challenges with respect to purity and consistency, leading to limited use. Recent studies have renewed interest in lysostaphin due to its effective antibiofilm activity, showing promise for treating staphylococcal infections, despite it not being a new antibiotic (Johnson et al., 2019). However, its immunogenic nature leads to rapid neutralization by the host immune system, thereby compromising efficacy and posing potential risks to patient safety. The development of deimmunized lysostaphin offers a promising strategy to circumvent human immune surveillance, thereby facilitating highly effective repeated dosing (Zhao et al., 2020). Moreover, compounds with completely new modes of action are being isolated from bacteria, although they have not yet reached the final stages of development. For example, quorum quenching enzymes, such as N -acyl homoserine lactones (AHLs) from bacteria, disrupt bacterial communication and biofilm formation. These innovative approaches provide exciting avenues for developing new treatments to combat antibiotic resistance.

### 2.3 Antibiofilm and innovative strategies

The protective environment of biofilms can significantly reduce the efficacy of antibiotics that would otherwise be successful against planktonic bacterial cells. Traditional minimum inhibitory concentration (MIC) tests often fail to capture this complexity in antibiotic resistance. Therefore, it is important to incorporate biofilm-specific measures like minimum biofilm inhibitory concentration (MBIC) and minimum biofilm eradication concentration (MBEC), which more accurately assess the effectiveness of treatments against biofilms (Thieme et al., 2019). This underscores the necessity for healthcare professionals to consider the unique challenges posed by biofilms in ODRIs and to explore enhanced therapeutic strategies, such as combination therapies. For instance, rifampin, also derived from *Streptomyces*, is a well-known biofilm-active antimicrobial that is effective in penetrating biofilms and targeting dormant bacteria (Zimmerli and Sendi, 2019). Similarly, ciprofloxacin, renowned for its efficacy against Gram-negative infections, also demonstrates significant antibiofilm properties, making it another valuable treatment option for combating Gram-negative ODRIs (Shariati et al., 2022). In response to the diminishing effectiveness of traditional antibiotics, optimization strategies have been reported; one of these is based on the seesaw effect. The seesaw effect describes the phenomenon where the resistance to glyco- and lipopeptide antibiotics increases while the resistance to β -lactam antibiotics decreases, and vice versa (Aunon et al., 2022). This is a specific example of the broader phenomenon of collateral sensitivity. Collateral sensitivity occurs when resistance to one antibiotic enhances the susceptibility to a second one. This is a relatively common phenomenon, and these relationships are summarized in collateral sensitivity networks. These networks inspire collateral sensitivity drug-cycling approaches and emphasize the importance of alternating or combining these medications to impede the adaptive capabilities of bacteria (Imamovic and Sommer, 2013).

**Table 1:** Novel natural product derivatives from microorganisms and fungi that inhibit antibiotic-resistant bacteria for use in humans.

Class	Drug name	Phase	Target	Effect	New class?	New target?	Potential indication(s)
Aminoglycoside	Plazomicin	Approved 2018	Ribosome 30S subunit	Blocks protein synthesis	No	No	CRE
Carbapenem	Tebipenem	3	Penicillin-binding proteins that catalyze peptidoglycan cross-linking	Blocks cell wall biosynthesis	No	No	ESBL-producing Enterobacterales
β -Lactam–siderophore hybrid	Cefiderocol	Approved 2019					*A. baumannii*, *P. aeruginosa*
Pleuromutilin	Lefamulin	Approved 2019	Ribosome 50S subunit	Blocks protein synthesis	No	No	MRSA
Lipoglycopeptide	Oritavancin and dalbavancin	Approved 2014	Bacterial cell wall synthesis	Blocks peptidoglycan building process	No	No	MRSA, enterococci, streptococci
Fusidane	Fusidic acid	3	Elongation factor 3	Blocks protein synthesis	No	No	MRSA
Tetracycline	Eravacycline	Approved 2018	Ribosome 50S subunit	Blocks protein synthesis	No	No	MRSA, ESBL-producing Enterobacterales
Antimicrobial peptide mimic	Murepavadin	3	LptD	Blocks outer-membrane biosynthesis	Yes	Yes	ESBL-producing Enterobacterales, *P. aeruginosa*
Defensin mimetic	Brilacidin	2	Cell membrane	Disrupts cell membrane	Yes	No	MRSA

The abbreviations used in the table are as follows: CRE – carbapenem-resistant Enterobacterales; ESBL – extended-spectrum β -lactamase; *A. baumannii* – *Acinetobacter baumannii*; *P. aeruginosa* – *Pseudomonas aeruginosa*; LptD – lipopolysaccharide transport protein D; MRSA – methicillin-resistant *Staphylococcus aureus*;.

## 3 Antimicrobials derived from viruses

### 3.1 Bacteriophage therapy

**Figure 2:**
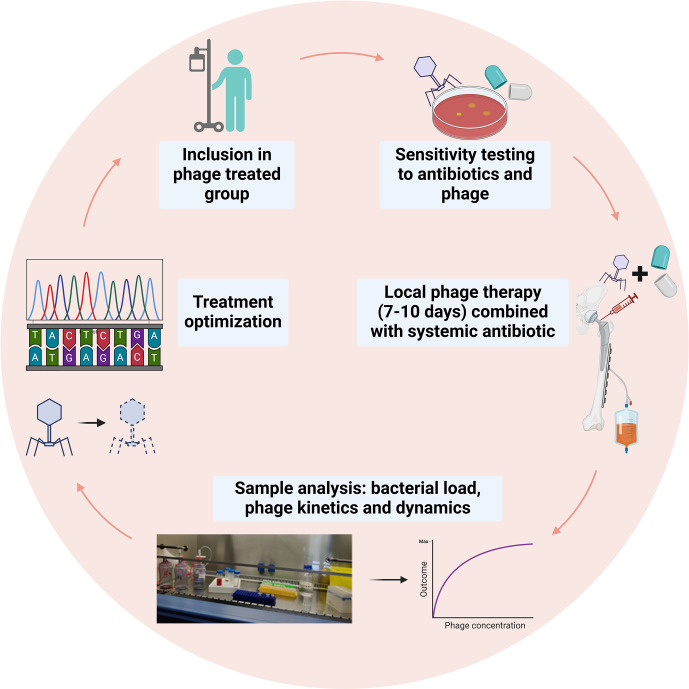
Proposed model of phage therapy for ODRIs from the PHAGEFORCE study (Onsea et al., 2021). Figure adapted from Bessems et al. (2024).

Bacteriophages, or “phages”, are highly specific viruses that are known to be natural enemies of their host bacteria. The use of phages for the treatment of bacterial infections is not a novel concept and has been applied since the start of the 20th century (Abedon et al., 2011). Phages and bacteria have coevolved over vast time spans, developing complex interactions that enable phages to effectively target and lyse specific bacterial cells while bacteria, in turn, evolve various defense mechanisms. Key properties of phages that contribute to their potential as a therapeutic option include their specificity to target bacteria, their ability to multiply at the site of infection and their minimal impact on the host's natural flora. The efficacy of phage therapy has been investigated in a recent systematic review, which showed that phage therapy is generally well tolerated in clinical settings for difficult-to-treat infections (Uyttebroek et al., 2022). However, due to the heterogeneity of study designs and administration protocols, drawing conclusive findings has been challenging. To address these concerns, a Belgian framework has developed the PHAGEFORCE study, which aims to implement phage therapy in different medical disciplines, using a standardized and multidisciplinary approach (Fig. 2; Onsea et al., 2021). To fully harness the potential of phage therapy, several challenges need to be addressed. Table 2 summarizes these challenges and the innovative methods being developed to overcome them. Immunogenicity is a significant limitation of phage therapy. The human immune system can potentially neutralize phages before they exert their therapeutic effect (Dabrowska, 2019). The development of these antibodies, however, exhibits interindividual variability and may be influenced by factors such as the frequency and duration of phage exposure. Studies have demonstrated that the antibody levels do not exhibit a significant correlation with the outcome of phage therapy (Lusiak-Szelachowska et al., 2017), indicating that additional factors may play a role in determining the therapy's efficacy. For the successful adoption of phage therapy in clinical practice, robust methods to monitor its efficacy are essential. Currently, phage titers can be detected from surgically retrieved tissue using the double-layer agar method to identify active phages (Kropinski et al., 2009). Additionally, polymerase chain reaction (PCR) can be employed to detect inactive phages (Acs et al., 2020). These techniques are critical in assessing the presence and activity of phages within treated tissues.

**Table 2:** Challenges to innovations in phage therapy and potential methods to overcome them.

Challenges in phage therapy	Method
Narrow host range	Application of phage cocktail
Emergence of phage-resistant bacteria	Phage cocktail – combination of antibiotic and phage
Less antibiofilm activity	Evolution of phage – genetically engineered phages
Immune-mediated neutralization	Encapsulation into biomaterials
Standardization and unknown genes risk	Development of phage-derived protein
Lack of randomized controlled trials (RCTs)	Increased investment in clinical research and conducting RCTs

### 3.2 The synergistic effect of bacteriophages and antibiotics

Phages can demonstrate heightened effectiveness against bacteria when used in conjunction with antibiotics. This synergy, known as phage–antibiotic synergy (PAS), is characterized by a mutual enhancement between phages and antibiotics, where their combined impact surpasses the effect of either treatment alone, potentially offering a more potent and effective approach to combating bacterial infections (Comeau et al., 2007). The phenomenon was attributed to the increased biomass and biosynthetic potential of bacteria in the presence of antibiotic concentrations high enough to inhibit cell division but not to cause cell death, resulting in a larger burst size and quicker spread of phages (Kamal and Dennis, 2015). This effect is observed with some phages and antibiotics, although not universally across all combinations, as the interactions can vary depending on the specific types of phages, antibiotics and bacterial strains involved. The clinical exploitation of PAS has two additional benefits: (1) phage can expose biofilm-embedded bacteria to antibiotics and (2) phage resistance in bacteria may reduce their fitness, making them more vulnerable to antibiotics (Chan et al., 2016). In a literature review aimed at understanding the implications of the development of phage resistance, 17 out of 22 articles showed an association between phage resistance costs and virulence reduction (Oechslin, 2018). However, phage–antibiotic antagonism has also been described in the literature, resulting in the reduced efficacy of combined treatment compared with phages or antibiotics individually (Diallo and Dublanchet, 2022). Whether a combination of phages and antibiotics will yield synergistic or antagonistic effects hinges on their respective mechanisms of action. In vitro studies have demonstrated antagonism for antibiotics that inhibit bacterial RNA polymerase (e.g., rifampin) or ribosomes (e.g., tobramycin), both of which are essential for phage replication (Lusiak-Szelachowska et al., 2022). The timing of application of phages and antibiotics also seems to be an important factor. To achieve optimal biofilm eradication outcomes and antibiotic resistance profiles, it has been recommended to delay antibiotic administration after phage pretreatment (Kumaran et al., 2018). This strategic delay ensures a more effective biofilm disruption and enhances bacterial reduction by allowing the phages to proliferate within the biofilm matrix, thereby increasing phage densities and priming the environment for deeper antibiotic penetration. Nonetheless, the success or failure of phage–antibiotic combination therapy is still in a state of immaturity, given that the optimal formulation of phage and antibiotic involved in synergy remains unclear, and the order of the phage–antibiotic staggered treatment needs to be addressed.

The application of a controlled-release system presents a promising strategy to address these uncertainties. By employing a controlled-release mechanism, the precise and controlled delivery of both phages and antibiotics can be facilitated. This approach allows for meticulous regulation of the timing and dosing of these agents, potentially optimizing their interaction for a more pronounced and sustained therapeutic impact. Several studies have demonstrated the potential of delivery systems in the context of phage application combined with antibiotics (Chen et al., 2023). These investigations have explored innovative methods for delivering both phages and antibiotics, aiming to effectively enhance phage–antibiotic combination therapy. Refinement of these systems can potentially lead to improved treatment outcomes, reduced resistance and enhanced clinical applicability in combating bacterial infections. While these systems have shown promise in preclinical studies, they have not yet been implemented in clinical settings.

### 3.3 Bacteriophage-derived proteins

Classic antibiotics do not directly affect the biofilm matrix, and their diffusion into biofilms is sometimes limited. Phage-derived proteins, such as endolysins, may provide an alternative therapeutic approach for ODRIs based on the mechanism of action of lytic phages (Sendi and Ferry, 2022). Endolysins are phage-derived enzymes with hydrolase activity that cleave various conserved peptidoglycan chemical bonds to break down the peptidoglycan layer and release phage progeny from the host bacterium (Drulis-Kawa et al., 2015). Endolysins can destroy Gram-positive bacteria including the surrounding biofilm matrix when applied externally, as these bacteria lack an outer membrane. In addition, phage lysins have a broader activity spectrum than phages, making them interesting antimicrobial candidates. For example, lysins purified from phages targeting *S. aureus* can also kill coagulase-negative staphylococci, which phages alone cannot do (Watson et al., 2019). Lysins have been shown, in numerous preclinical studies, to be effective with respect to killing both planktonic and biofilm bacteria, and combining lysins with classical antibiotics reduces bacterial numbers on bone and implants (Karau et al., 2022).

Currently, three lysins, P128 (StaphTAME), CF-301 (exebacase) and SAL200 (tonabacase), are in development at different clinical stages for similar indications including *S. aureus* bacteremia. CF-301 was applied locally and administered in combination with systemic daptomycin and linezolid, demonstrating its potential as a salvage therapy to enhance the effectiveness of antibiotic treatment and prevent functional loss in patients with staphylococcal PJI of the knee, with the clinical outcome being decidedly favorable for all patients (Ferry et al., 2021). A phase-II clinical trial for SAL200 (Intron Biotechnology, 2019) assessed its efficacy and safety specifically in *S. aureus* bacteremia but terminated prior to completion. However, two patients who received SAL200 developed severe side effects (pneumonia or respiratory failure), and two patients in the placebo group experienced serious side effects (cardiac disorder due to acute infection or type-2 respiratory failure). The reasons for these side effects are unclear, as detailed results have not been published. It remains uncertain whether these were related to endotoxin levels, patient comorbidities, the infection or other factors. Currently, the specific phases of clinical trials for endolysins in the context of ODRIs are not explicitly outlined in the available literature.

However, due to their mechanism of action, these endolysins are limited to killing Gram-positive bacteria. Gram-negative pathogens are more difficult to treat with phage lysins due to the presence of a protective outer membrane. Hence, several research groups are actively working on the development of phage peptidoglycan hydrolases (PGHs) to specifically target Gram-negative pathogens (Ramesh et al., 2022). One such approach is the use of artilysins, which are engineered endolysin-based proteins developed by Lysando AG. Artilysins consist of endolysins that are recombinantly fused to an outer-membrane-permeabilizing peptide. Recently, on the basis of the huge diversity of outer-membrane proteins, although also considering cell-wall-binding and enzymatic-activity domains that determine the antibacterial activity, Gerstmans et al. (2023) developed the VersaTile technique. This technique facilitates the construction and screening of a combinatorial library of modular, engineered lysins. Although these engineered lysins have shown promising antibacterial properties against Gram-negative pathogens in laboratory studies, it is crucial to conduct registered clinical trials to provide robust evidence and support these claims in a clinical setting. To provide a clearer understanding of the advantages and disadvantages of different therapeutic approaches, Table 3 compares antibiotics, phage therapy and endolysins.

**Table 3:** A comparison of the advantages and disadvantages of antibiotics, phage and endolysins.

Consideration	Antibiotic therapy	Phage therapy	Endolysins
Specificity	Low	High	Intermediate
Ease of discovery	Challenging	Simple	Challenging
Mechanism of action	Inhibits key bacterial functions, like cell wall, protein or DNA synthesis	Infects specific bacteria, replicates inside and causes cell lysis	Breaks down peptidoglycan in bacterial cell walls, causing cell lysis
Side effects	Moderate–high	Minimal side effects	Minimal side effects
Resistance	Frequent development	Develops, varies by bacterium	Low
Antibiofilm activity	Limited effectiveness	Effective with antibiotics	Effective on Gram-positivebiofilms
Impact on human microbiome	High impact	Low impact	Low impact
Kinetics	Depends on concentration	Self-amplifying	Rapid bacterial disruption
Clinical validation	Many trial studies	Limited but growing number of clinical trials	Emerging clinical evidence
Scalability of production	Well-established, scalable manufacturing processes	Production challenging,requires controlledbioprocessing	Scalable via recombinant DNA technology but requires optimization

## 4 Antimicrobials derived from plant sources

Plants have long served as a vital source of drugs and alternative medicine in the battle against diseases, dating back to ancient times. They offer a wealth of valuable secondary metabolites, including quinones, tannins, terpenoids, alkaloids, flavonoids and polyphenols. These compounds serve as defense mechanisms for plants against microorganisms, insects and herbivores. Nevertheless, since the arrival of antibiotics in the 1950s, the use of plant derivatives as antimicrobials has been nonexistent. However, contemporary researchers are reigniting interest in plant extracts as potential medicines, considering them as viable alternatives to conventional antibiotics (Subramani et al., 2017).

The formulation of plant-derived compounds has demonstrated potential effectiveness against AMR when used in conjunction with traditional antibiotics. There is existing evidence supporting the enhanced activity of conventional antibiotics through synergistic interactions with plant-derived compounds. For instance, the combination of curcumin and erythromycin demonstrated a significant suppression of bacterial growth compared with their respective monotherapies in a rat model of MRSA-induced osteomyelitis (Zhou et al., 2017). Allicin, in another study, inhibited biofilm formation and augmented the bactericidal effect of vancomycin in a rabbit model of PJI caused by *S. epidermidis* (Zhai et al., 2014). These findings suggest promising avenues for developing novel treatment strategies against ODRIs by harnessing the complementary actions of natural compounds and antibiotics. Moreover, plant-derived compounds show promise in restoring the clinical application of older antibiotics by enhancing their potency, thereby mitigating the development of resistance. The primary mechanism leading to MDR is the overexpression of efflux pump systems, which extrude antibacterial molecules from bacterial cells, reducing their concentrations below effective levels. Several types of plant-derived extracts have been identified to block efflux pumps in both Gram-negative and Gram-positive bacteria, offering the potential to restore antibiotic efficacy. This allows antibiotics to attain sufficient concentrations inside bacteria for a bactericidal effect (Seukep et al., 2020). However, further research, both in vitro and in vivo, is essential to identify active and nontoxic antimicrobial phytochemicals for effective application.

A multicenter retrospective study discovered that the combination of medicinal plants with conventional antibiotic therapy proved more effective in treating drug-resistant enterobacterial infections compared with conventional monotherapy (Cai et al., 2017). The herbal medicines employed in this study, such as “Flos Lonicerae”, “Radix Angelicae Sinensis”, “Radix Astragali seu Hedysari”, “Radix Paeoniae Rubra”, “Radix Rehmanniae Recens” and “Fructus Gardeniae”, were found to offer a more balanced immune response compared with the excessive inhibition observed with antibiotic monotherapy. It is worth noting that this study is retrospective and lacks specific treatment details, posing challenges for replication by other studies. Bu Zhong Yi Qi Tang, a traditional Chinese herbal medicine, is commonly used to treat severe weakness, loss of appetite and indigestion in elderly patients as well as to prevent opportunistic infections in infection-prone individuals (Kohno et al., 2021). This formulation consists of *Astragalus* root, ginseng root, white *Atractylodes* rhizome, Chinese *Angelica* root, processed licorice root and *Cimicifuga* rhizome, primarily derived from plant roots. A RCT demonstrated that the Bu Zhong Yi Qi Tang group showed significant wound-healing progression and a decrease in the total DESIGN-R score, compared with the control group which showed minimal progress (Akita et al., 2019). The comprehensive understanding of plant extract composition poses a significant challenge due to the multitude of components, making interpretation difficult. While achieving standardization, stability and quality control is challenging, the opportunity to explore a large quantity of unexplored compounds may renew interest in medicinal plants.

## 5 Antimicrobials derived from other sources

### 5.1 Maggot therapy

It has been suggested that animals in polluted and germ-infested environments have developed antibacterial properties that protect them against pathogenic microorganisms. Maggot therapy (MT) is a type of biotherapy that involves the introduction of live, disinfected maggots into nonhealing skin and soft-tissue wounds. A large amount of clinical evidence supports MT as an effective treatment against AMR and to improve wound-healing rates (Malekian et al., 2019). These maggots secrete enzymes that break down necrotic tissue, which they then ingest, effectively cleaning the wound. MT has at least two confirmed beneficial effects when applied to wounds, including the removal of necrotic tissue and pathogenic bacteria. For example, a meta-analysis of several RCTs reported that wounds treated with MT have faster wound debridement, granulation tissue development and wound surface area reduction (Mohd Zubir et al., 2020). However, concerns about discomfort, including pain and the psychological aversion to the use of maggots, have restricted their use. Nonetheless, for most patients, the drawbacks of MT pale in comparison to its remarkable efficacy in treating even the most recalcitrant wounds. Researchers continue to explore the mechanisms by which maggots help heal wounds. Currently, MT is recognized and regulated as a medical device in several countries, including the United States and parts of Europe. Its application remains limited but is approved under specific conditions, primarily for wounds that do not respond to conventional treatments. As research progresses, further clarification and expansion of its clinical indications and regulatory approval are anticipated.

### 5.2 Animal sources

Antimicrobial polypeptides produced by animals, such as honeybees, fruit flies and frogs, have shown a broad range of antibacterial properties against MDR bacteria in vivo. Innate defense regulator peptide 1018 (IDR-1018) is a synthetic 12-amino-acid derivative of bovine neutrophils (Wieczorek et al., 2010). IDR-1018 can directly kill bacteria; moreover, it can modulate the differentiation and activation of macrophages and neutrophils, thereby regulating their production of chemokines and cytokines. Although these peptides are effective, they often have serious and unwanted side effects, such as cytotoxicity, allergenicity and immune system modulation, which can lead to inflammation and other adverse reactions (Pang et al., 2019). The antimicrobial metabolites from animals often display unspecific toxicity to cells, so their devastating power requires modulation. Currently, there are no RCTs for these animal-derived antimicrobial peptides, highlighting the need for further research to validate their efficacy and safety in clinical settings. In the future, these antimicrobial agents will need improvement and/or novel formulations to become less toxic, more bioavailable and useful in the biomedical field.

### 5.3 Marine sponges

Marine sponges produce a myriad of bioactive secondary metabolites. Two classes of these metabolites are most promising for evolutionary robust antimicrobial strategies – the terpenoids and the pyrrole–imidazoles – as these compounds have been shown to modulate biofilm formation without disrupting the growth or killing the bacteria (Dieltjens et al., 2020). While not exclusive to sponges, terpenes and their derivatives represent one of the most potent and diverse groups of molecules. Terpenes exhibit a remarkable structural diversity resulting from the modification of isoprene subunits and are esteemed for their extensive biological activity, spanning from antifouling agents to cancer therapeutics with antiproliferative properties (Ebada et al., 2010). Pyrrole–imidazoles from marine sponges have inspired the creation of effective antibiofilm compounds using the 2-aminoimidazole moiety. These compounds show strong activity against Gram-positive bacteria, Gram-negative bacteria and the yeast *Candida albicans*, with efficacy influenced by side-chain modifications (Gomes et al., 2020). Inhibiting bacterial cooperation in biofilms is a robust strategy against resistance. Additionally, a 2-aminoimidazole coating on titanium has shown antibiofilm effectiveness in mouse infection models and demonstrated stability and biocompatibility in rabbit bone models (Coppola et al., 2021).

### 5.4 Mineral sources

Silver and copper are crucial trace elements with potent antimicrobial properties, especially valuable in combating ODRIs. These metals exert their effects primarily through the disruption of bacterial cellular processes and inducing oxidative stress within pathogens, leading to bacterial death. Silver has been extensively used in medical applications due to its broad-spectrum antimicrobial capabilities. It can be integrated into coatings on medical devices and implants or incorporated into wound dressings to reduce the risk of infection (Savvidou et al., 2020). The ionic form of silver interferes with bacterial DNA and protein functions, effectively halting their ability to multiply and spread (Mijnendonckx et al., 2013). Copper, although less commonly used than silver, also holds significant antimicrobial potential. It can be alloyed with other metals in implants or utilized in surface coatings. Copper ions damage bacterial cell membranes and disrupt essential cellular enzymes, preventing the bacteria from performing vital metabolic functions (Bisht et al., 2022). These minerals are especially useful against drug-resistant bacteria in ODRIs. However, the sustainability of these compounds is limited due to potential environmental toxicity, bacterial resistance development and the high cost of continuous usage. Ongoing research aims to improve their delivery methods and reduce side effects.

## 6 Conclusions and future perspectives

This narrative review provides a descriptive overview of the multifaceted realm of naturally occurring antimicrobials and their pivotal role in addressing the escalating challenges posed by ODRIs. These infections, notably PJIs and FRIs, present significant clinical challenges due to the prevalence of AMR and the complex nature of biofilm formation on orthopedic implants. The future of ODRI treatment lies not only in the discovery of new antimicrobial agents but also in the innovative application and combination of existing ones. While challenges remain in terms of clinical application and regulatory approval, the potential of these natural antimicrobials to revolutionize the treatment of ODRIs cannot be overstated. As research progresses, it is imperative to continue exploring these natural resources, with a focus on enhancing their efficacy, reducing toxicity and improving bioavailability, thereby offering new hope in the battle against one of the most daunting challenges in modern medicine.

## Data Availability

No data sets were used in this article.
